# Genetic and Molecular Analysis of Wild-Derived Arrhythmic Mice

**DOI:** 10.1371/journal.pone.0004301

**Published:** 2009-01-28

**Authors:** Tsuyoshi Watanabe, Tohru Suzuki, Akira Ishikawa, Yuki Yokota, Hiroki R. Ueda, Rikuhiro G. Yamada, Hajime Tei, Saki Imai, Shigeru Tomida, Junya Kobayashi, Emiko Naito, Shinobu Yasuo, Nobuhiro Nakao, Takao Namikawa, Takashi Yoshimura, Shizufumi Ebihara

**Affiliations:** 1 Division of Biomodeling, Graduate School of Bioagricultural Sciences, Nagoya University, Nagoya, Japan; 2 Department of Infectious Disease, Hamamatsu University School of Medicine, Hamamatsu, Japan; 3 Division of Applied Genetics and Physiology, Graduate School of Bioagricultural Sciences, Nagoya University, Nagoya, Japan; 4 Laboratory for Systems Biology, Center for Developmental Biology, RIKEN, Hyogo, Japan; 5 Functional Genomics Subunit, Center for Developmental Biology, RIKEN, Hyogo, Japan; 6 Research Group of Chronogenomics, Mitsubishi Kagaku Institute of Life Sciences, Tokyo, Japan; Duke University, United States of America

## Abstract

A new circadian variant was isolated by screening the intercross offspring of wild-caught mice (*Mus musculus castaneus*). This variant was characterized by an initial maintenance of damped oscillations and subsequent loss of rhythmicity after being transferred from light-dark (LD) cycles to constant darkness (DD). To map the genes responsible for the persistence of rhythmicity (circadian ratio) and the length of free-running period (τ), quantitative trait locus (QTL) analysis was performed using F_2_ mice obtained from an F_1_ cross between the circadian variant and C57BL/6J mice. As a result, a significant QTL with a main effect for circadian ratio (*Arrhythmicity*; *Arrh-1*) was mapped on Chromosome (Chr) 8. For τ, four significant QTLs, *Short free-running period* (*Sfp-1*) (Chr 1), *Sfp-2* (Chr 6), *Sfp-3* (Chr 8), *Sfp-4* (Chr 11) were determined. An epistatic interaction was detected between Chr 3 (*Arrh-2*) and Chr 5 (*Arrh-3*). An *in situ* hybridization study of clock genes and mouse *Period1::luciferase* (*mPer1::luc*) real-time monitoring analysis in the suprachiasmatic nucleus (SCN) suggested that arrhythmicity in this variant might not be attributed to core circadian mechanisms in the SCN neurons. Our strategy using wild-derived variant mice may provide a novel opportunity to evaluate circadian and its related disorders in human that arise from the interaction between multiple variant genes.

## Introduction

Circadian rhythms are fundamental properties of organisms living on earth and numerous physiological and behavioral functions are under the control of the circadian clock. In mammals, the circadian pacemaker resides in the suprachiasmatic nucleus (SCN), in which the self-sustained oscillation is generated by the interaction of a set of activated clock genes [1]. Over the past decade, multiple clock genes have been identified, and it is now generally accepted that the generation of circadian rhythms is based on transcription-translation feedback loops comprising the activation of multiple clock genes. Meanwhile, several genome-wide genetic analyses have demonstrated that many genes other than clock genes are involved in the circadian clock function [2]–[7]. In addition, recent studies analyzing genome-wide gene expression patterns have revealed that many genes in the SCN and peripheral organs oscillate with circadian rhythmicity [8]–[10]. Thus, it is likely that a variety of genes are involved in refining and amplifying the core circadian oscillation and maintaining the normal temporal physiology of the complex circadian system.

It is now appreciated that disturbed circadian rhythms are associated with many mental and physiological disorders, but little is known about how circadian rhythms are implicated in the onset of these disorders. To study these issues, appropriate model mice that show aberrant circadian rhythms are substantially useful. So far, several circadian mutant mice, most of which were created by mutagenesis or gene targeting, have been used for the model system in human circadian and its related disorders [11]. However, it appears that most of human diseases result from deleterious combinations of polymorphic alleles with small effects on the phenotypes and the diseases elicited by single gene defects are fairly rare.

To establish model systems useful for circadian and its related disorders in human, we attempted to obtain variants from wild-caught mice (*Mus musculus castaneus*) because natural populations of organism conserve genetic diversity (the subset of polymorphic loci) that give rise to phenotypic variation (i.e., many naturally occurring variant genes, including harmful and beneficial genes, are conserved in wild populations). In *Drosophila*, for example, numerous variants have been isolated from wild populations [12]. Because commonly used laboratory mice are known to have originated from a limited founder population in a few laboratories [13], [14], new genetic variants affecting circadian rhythms, which we cannot find in commonly used laboratory mice, might be obtained from wild mouse population.

In this attempt, we successfully isolated mice in which behavioral circadian rhythms were abolished under conditions of constant darkness (DD). We designated this variant the “circadian variant.” In the present study, therefore, we initially performed a quantitative trait locus (QTL) analysis to map the QTLs responsible for the persistence of circadian rhythmicity and the free-running period (τ) in this circadian variant. We then undertook sequencing analysis to search the candidate genes for QTLs. Finally we investigated how the expression of clock genes was altered in the SCN of these mice.

## Materials and Methods

### Animals and housing

Sixteen adult wild mice (*Mus musculus castaneus*) were captured at several locations in the vicinity of University of the Philippines, Los Banos, in June 1994 and shipped to our laboratory. A pair of the mice captured in the market (a female mouse in Bayan market and a male mouse in New market) were bred to produce the experimental progenies. Three female mice obtained from the paired mice were backcrossed to a wild-caught male mouse, producing a total of 24 backcross progeny. These mice were screened for locomotor activity rhythms and we identified two individuals (one male and one female) that exhibited abnormal patterns of activity rhythms: a large positive phase angle under 12 h light-12 h dark (LD 12∶12) cycles and spontaneous rhythm splitting under constant darkness (DD) conditions. These mice were mated and their offspring were tested for activity rhythms. Of these mice, some exhibited an abnormal activity rhythm pattern similar to that of their parents. One abnormal male was backcrossed to the mother, and among the progeny of this mating we discovered one mouse that became arrhythmic under DD conditions. This variant mouse was crossed to an abnormal littermate exhibiting a significant positive phase angle. In this intercross, we obtained several circadian variants that were used for subsequent breeding to maintain this phenotype. Because reproductive efficiency was reduced during the intercross of the wild mice, one circadian variant was crossed to a C57BL/6J mouse (CLEA Inc., Tokyo, Japan) and, subsequently, an F_1_ female offspring was backcrossed to the parental circadian variant, producing one male and one female circadian variant. These mice were mated and consecutive sib mating was continued for 7 generations. For genetic analysis, 18 F_1_ and 236 F_2_ progeny (males, *n* = 125; females, *n* = 111) obtained from the cross between one male circadian variant from the colony of 7 generations and C57BL/6J mice were analyzed.

During the experiment, the mice (50–80 days old at the beginning of the experiment) were individually housed in a cage equipped with a running wheel (diameter, 10 cm; width, 5 cm) in a light-tight box. The light intensity inside the cages was 300–500 lux. The room temperature was maintained at 23±2°C throughout the experiment. Food and water were available *ad libitum*. In all the experiments, the animals were treated in accordance with the guidelines of Nagoya University.

### Behavioral analysis

**Figure 1:**
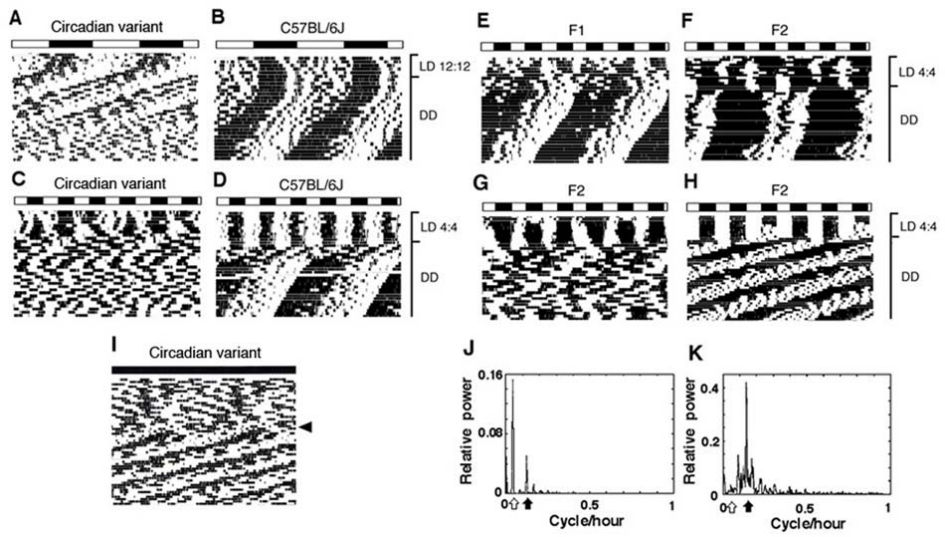
Activity profiles of wild-derived circadian variant, C57BL/6J mice, and their F_1_, F_2_ offspring. Representative locomotor activity rhythms (double-plotting) of the circadian variant (A and C) and C57BL/6J (B and D) mice maintained under LD 12∶12 and LD 4∶4, and then transferred to DD. One F_1_ and three F_2_ mice with different activity patterns are shown in E–H. The arrhythmic mouse (I) received a 6-h light pulse (indicated by an arrow head) under DD (see circadian rhythms recovered). Light and dark cycles are represented by white and black bars above each record. The results of fast Fourier transform analysis applied to the actograms of (F) and (G) for the final 15 days are shown in (J) and (K), respectively. Open and Closed arrows represent the maximum peaks of relative power in the circadian (18–30 h) and ultradian (1–18 h) range, respectively.

Wheel-running activity was continuously monitored and recorded by a computer system (The Chronobiology Kit; Stanford Software Systems, CA). The locomotor activity rhythms were measured either under LD 12∶12 or LD 4∶4 conditions for 2 weeks and subsequently under DD conditions for 7 weeks. To assess the persistence of circadian rhythmicity, fast Fourier transform analysis in the MatLab environment (ClockLab; Actimetrics, Evanston, IL) was performed at 15-days intervals in DD. The power spectral densities for frequencies ranging from 0 to 1 cycle/h were determined and normalized to a total power of 1.0 [15]. The peaks of the relative power in the circadian (18–30 h; 1/18–1/30 cycle/h) and non-circadian (1–18 h; 1/1–1/18 cycle/h) ranges were determined for each animal; the rhythm with a period ranging from 18 to 30 h was arbitrarily assigned as the circadian rhythm (Fig. 1J, K). The rhythmicity was evaluated using the following method. The ratio (%) for the relative power of the largest circadian peak to the sum of the largest circadian and non-circadian peak values was calculated at 15-d intervals (we term this value the circadian ratio). The minimum ratio was defined as an individual phenotype. If the ratio exceeded 50%, the activity pattern was judged as circadian, and if not as non-circadian. In DD conditions, τ, which was determined by a chi-square (χ^2^) periodogram analysis, was calculated at 15-d intervals, and the lowest value was used for QTL analysis because τ was positively correlated with the circadian ratio (Fig. S2). After being transferred from the LD 4∶4 to DD conditions, most of the circadian variants (*n* = 89) and some of the F_2_ mice (*n* = 10) immediately became arrhythmic; therefore, these mice were not included in the QTL analysis for τ. Daily activity counts under DD conditions were obtained using the Chronobiology Kit, taking a daily average for the 15-d interval in which the minimum circadian ratio was exhibited. The difference in phase angle between the onset of activity and the offset of the light was calculated based on the activity profile using the Chronobiology Kit. The time elapsed at a quarter of the maximum counts was defined as the onset of activity.

### Marker analysis

The microsatellite (*Mit*) markers used in this study were purchased from Research Genetics Inc. (Huntsville, AL). Since the size of marker alleles in wild-derived mice was unknown, hundreds of markers, which were selected on the basis of allelic information on the CAST/Ei and C57BL/6J strains from the Mouse Genome Database on all chromosomes (Chrs), were tested using the parents and F_1_ mice. Finally, a total of 271 informative markers distributed throughout all the Chrs could be reliably distinguished among the circadian variant, C57BL/6J, and the F_1_ mice (Table S1). In this study, alleles from the circadian variant and C57BL/6J mice were referred to as C and B, respectively. The genomic constitution of the parental variant male mouse was shown (Fig. S1). Marker linkage maps were constructed using the computer software Map Manager QTXb20 [16].

Genomic DNA was extracted from the tail of each mouse by the standard phenol∶chloroform method. Polymerase chain reaction (PCR) amplifications of the *Mit* markers were performed according to the PCR protocol described in our previous paper [17].

### QTL analysis

Initially, interval mapping using a least-squares regression method (Map Manager QTXb20) was performed on 236 F_2_ hybrids using 60 *Mit* markers (2 to 4 markers per chromosome). When suggestive or significant QTLs were detected, further analysis using additional markers (total 104) flanking potential QTLs was conducted. The likelihood of odds (LOD) scores at the genome-wide significant (5%) and suggestive (10%) levels were determined by 1000 runs of a permutation test [18]. The LOD scores for the circadian ratio at the genome-wide 5% and 10% significant levels were 3.65 and 2.09, and 3.43 and 2.04 for τ, respectively.

The epistatic interaction analysis was performed as described by Ishikawa et al. [19]. To declare the significant epistatic QTLs, two tests were conducted. First, the significance of the overall effect was established by 1000 runs of a permutation test using Map Manager QTXb20 and then, if the overall effect exceeded the genome-wide 5% significance level, the interaction was tested using approximate genome-wide thresholds obtained as described by Knott et al. [20].

The parental male mouse retains a heterozygous allele for loci (Fig. S1); therefore, we excluded the F_2_ mice produced by the B/C×B/B backcross from the QTL analysis using Map Manager QTXb20. Instead, we conducted QTL analysis for these loci with QTL Express. Using QTL Express, it is possible to analyze the combined populations of backcrosses and F_2_ crosses [21]. At the loci where the genotype of the parental variant male were B/B (Fig. S1), these markesr were excluded from the analyses using Map Manager QTXb20 and QTL Express.

The above QTL analysis is based on a one-QTL model. When two LOD peaks were observed on a chromosome, a two-QTL model was implemented using QTL Express. A statistical comparison between one- and two-QTL models was performed, as described by Wei et al. [22].

### Sequencing analysis of the candidate genes

Total RNA was extracted from the whole brains of circadian variants, C57BL/6J and CASP mice — the last of which had been established as an inbred strain by brother-sister mating from the same pair as founder mice of the circadian variant [23]— using TRIzol reagent (GIBCO BRL, Frederick, MD), then reverse transcription PCR was performed using SuperScript III First-Strand Synthesis System (Invitrogen, Carlsbad, CA). The CASP mice exhibited no obvious abnormalities in the circadian rhythms of locomotor activity under DD conditions. All nucleotide sequencing was performed bidirectionally by direct sequencing using a BigDye terminator v3.1 cycle sequencing ready-reaction kit (Applied Biosystems, Foster City, CA). We sequenced the coding regions of 69 genes located between *D8Mit205* (30 cM, 52.3 Mb) and *D8Mit249* (37 cM, 83.8 Mb) on Chr 8; these genes are likely to be expressed in the SCN (H.R. Ueda et al., unpublished observation).

### In situ hybridization

Arrhythmic mice were selected prior to the sampling. The mice maintained under LD 12∶12 for 2 weeks or DD for 1–2 weeks following transfer from LD were killed by decapitation and the brains were immediately removed. *In situ* hybridization was carried out according to Yoshimura et al. [24]. Antisense 45-mer oligonucleotide probes (*mClock*: nucleotides 642–686 of GenBank accession number AF000998; *mBmal1*: 1755–1799 of AB015203; *mPer1*: 3239–3283 of AB002108; *mPer2*: 242–286 of AF035830; and *mPer3*: 2301–2345 of AF050182) were labeled with [^35^S]dATP (New England Nuclear, Boston, MA) using terminal deoxyribonucleotidyl transferase (GIBCO-BRL). In the light-pulse experiment, mice were exposed to a 15-min light pulse (100 lux of fluorescent lamps) at circadian time (CT) 16 on the first day after transfer from LD 12∶12 to DD (16 h after transfer to DD; CT0 is defined as the time when lights would have come on under LD 12∶12). Samples were collected 90 min after the light pulse.

### mPer1::luc real-time monitoring

To know temporal changes of SCN physiology when the circadian variant became behaviorally arrhythmic, the mouse *Period1::luciferase* (*mPer1::luc*) real-time monitoring technique was used. For this, the variant mice were mated with *mPer1::luc* transgenic mice, in which the expression of luciferase is driven by a 6.7-kb genomic fragment of the mouse promotor (C57BL/6J) [25]. The F_1_ mouse was backcrossed to the parental variant and their offspring were screened for both behavioral rhythms and the *mPer1::luc* transgene. The mice that became arrhythmic under DD conditions and possessed *mPer1::luc* transgene were humanely sacrificed by cervical translocation and decapitation between either 0500 and 0600 or 1700 and 1800 (local time). Their brains were rapidly removed and placed in cold Hank's balanced salt solution (Invitrogen). Coronal sections (200-µm thick) of the SCN were prepared using a DTK-3000W vibratome. The SCN slices were placed on culture membranes (Millicell-CM, PICM030-50; Millipore, Bedford, MA) and cultured in 35-mm petri dishes with 1.2 ml of Dulbecco's modified Eagle's medium (Sigma, St. Louis, MO) supplemented with 3.5 g/l D-glucose, 10 mM HEPES, 25 U/ml penicillin, 25 µg/ml streptomycin, and 0.1 mM luciferin. We did not use any chemicals to stimulate the slice. Each dish was sealed using a coverslip and vacuum grease. These experiments were performed according to the methods previously described in Watanabe et al. [26]. Slice culture was performed at 36°C in a light-tight black box, and bioluminescence from the SCN was visualized using an Olympus BX microscope equipped with a Hamamatsu C4742-98-24NR ORCA camera. Images were acquired and analyzed using AquaCosmos software (Hamamatsu Photonics, Hamamatsu, Japan). The *mPer1::luc* bioluminescence rhythms from the whole SCN and individual SCN cells were measured during 0–60 h *in vitro*, and the distribution of the peak time for the individual SCN cells (30–40 cells per SCN slice) was measured.

### Statistical analysis

The differences between two groups were analyzed with a two-tailed Student's *t* test or the Mann-Whitney *U* test. Comparison among the multiple groups was performed using a one-way ANOVA. The correlation between the circadian ratio and τ was examined using Spearman's rank correlation test.

## Results

### Behavioral analysis of parental, F_1_, and F_2_ mice

Of the 52 circadian variant mice that were entrained to LD 12∶12 and transferred to DD, 28 mice (54%) lost and 24 mice (46%) maintained circadian rhythms of locomotor activity during 7 weeks (Table 1). There were no significant sex differences in the frequency of the mice with abolished circadian rhythms (*p*>0.5; Student's *t* test). To increase the ratio of arrhythmic mice for QTL analysis, we exposed the mice to an LD 4∶4 schedule before transferring them to DD; this is because SCN organization can be disrupted by abnormal LD cycles [26]. Indeed, the percentage of the arrhythmic phenotype dramatically increased (83%) and the circadian ratio decreased in both the arrhythmic and rhythmic phenotypes (Table 1). The arrhythmic state was not irreversible since a single light pulse could restore the rhythm (Fig. 1I). In addition, a few arrhythmic mice exhibited the spontaneous recovery of free-running rhythms without any specific treatments (data not shown). In the C57BL/6J mice, activity rhythms were divided into three components by this abnormal LD cycle and these animals exhibited short-term disturbed patterns after release into DD (Fig. 1B, D). The mean values of τ and daily activity counts in the circadian variant mice transferred from LD 4∶4 to DD were significantly lower than those in C57BL/6J mice (*p*<0.01; Student's *t* test).

**Table 1:** Summary of circadian parameters of parents, F1 and F2 mice.

Characteristics	Arrhythmic	Rhythimic	*p*-value
**Circadian variant (LD12∶12→DD)**			
Number	28	24	
Free-running period (hours)	22.70±0.16	22.61±0.10	n.s.^a^
Circadian ratio (%)	25.76±2.73	72.91±2.11	<0.0001
Daily activity in DD (counts/day)	14593±2977	13086±2409	n.s.
Phase angle difference (hours)	1.79±0.40	2.18±0.54	n.s.
**Circadian variant (LD 4∶4→DD)**			
Number	101	21	
Free-running period (hours)	22.36±0.12	22.55±0.13	n.s.
Circadian ratio (%)	20.24±1.27	65.1±2.09	<0.0001
Daily activity in DD (counts/day)	13461±955	13379±1029	n.s.
**C57BL/6J (LD 4∶4→DD)**			
Number		4	
Free-running period (hours)		23.28±0.09	-
Circadian ratio (%)		78.65±6.29	-
Daily activity in DD (counts/day)		42762±2682	-
**F1 (LD 4∶4→DD)**			
Number	4	14	
Free-running period (hours)	23.40±0.39	22.62±0.17	n.s.
Circadian ratio (%)	39.38±2.81	73.09±1.94	<0.0001
Daily activity in DD (counts/day)	46416±8411	33445±5229	n.s.
**F2 (LD 4∶4→DD)**			
Number	43	193	n.s.
Free-running period (hours)	22.64±0.11	22.81±0.05	n.s.
Circadian ratio (%)	26.83±2.20	77.89±0.84	<0.001
Daily activity in DD (counts/day)	37630±3495	44054±1709	n.s.

a not significant.

Free-running period of arrhythmic group was obtained only during 15-days intervals with the circadian ratio more than 50%.

**Figure 2:**
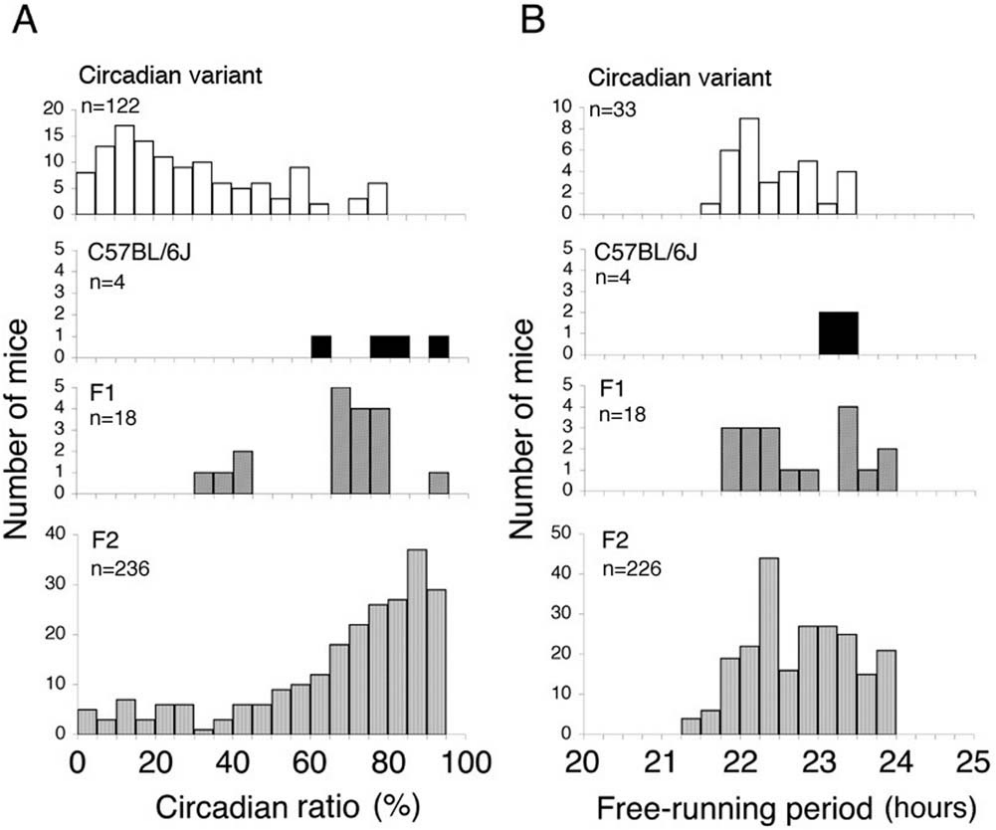
Frequency histograms of phenotypic values for parental, F_1_, and F_2_ mice. The distribution of phenotypic values of circadian ratio (A) and free-running period (B) are shown. The circadian ratio (%) is the relative power of the largest circadian peak to the sum of the largest circadian peak and non-circadian peak values. The free-running period (τ) was determined by a chi-square (χ^2^) periodogram analysis. Mean values of each phenotype for these mice are summarized in Table 1.

In the F_2_ mice, various patterns of circadian rhythm were observed, which is reflected in the wide distribution of the circadian ratio (Fig. 1F–H, Fig. 2A). τ was also widely distributed, encompassing the range of parents and F_1_ mice (Fig. 2B). It was generally observed that the shorter τ became, the less clear was the circadian rhythm; this was reflected in the significant positive correlation between the circadian ratio and τ in the F_2_ mice (ρ = 0.197, *p* = 0.003; Spearman's test) (Fig. S2).

### QTL analysis

**Figure 3:**
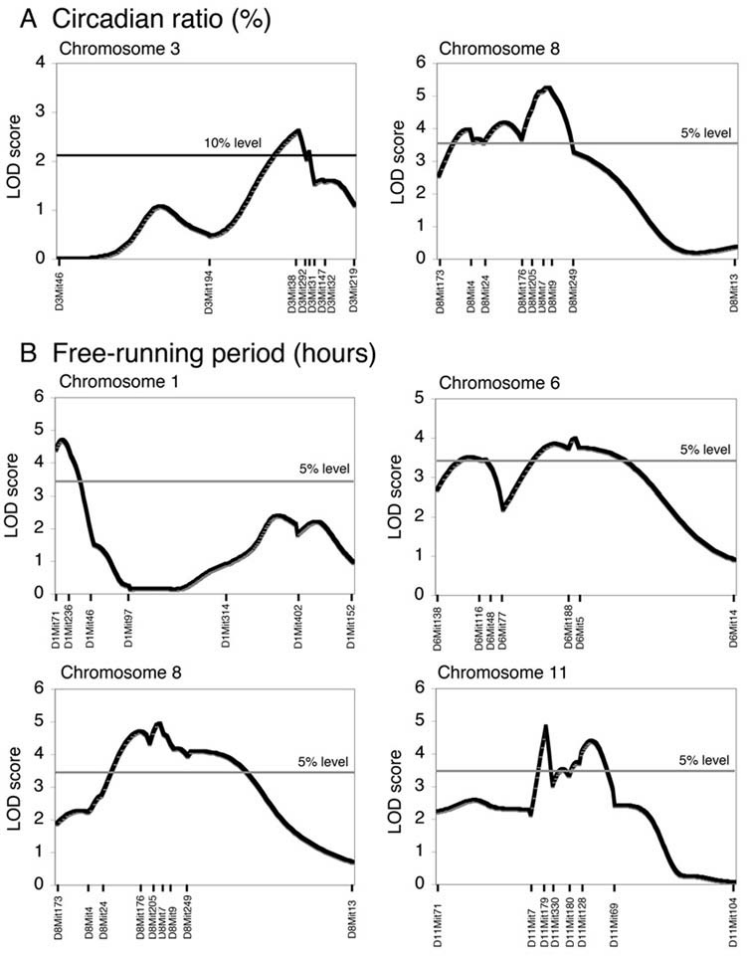
LOD score plots of QTLs with main effects. Results of interval mapping for the circadian ratio (A) and free-running period (B) using Map Manager QTXb17 software. The horizontal lines show the genome-wide 10%, 5% significance levels estimated by a permutation test. No statistical evidence for the presence of two QTLs was observed for these two traits.

To map the genes responsible for disorganized circadian rhythms, QTL analysis for the circadian ratio and τ was performed. Because there were no significant differences in the frequency of the mice with abolished circadian rhythm, both male and female data were combined to analyze. With regard to the circadian ratio, QTL analysis using Map Manager QTXb20 revealed a significant QTL (*Arrhythmicity*; *Arrh-1*, LOD 5.26) on Chr 8 (Fig. 3A, Table 2). Moreover, a suggestive QTL for the circadian ratio was mapped on Chr 3 (LOD 2.63). With regard to τ, four significant QTLs were mapped on Chr 1 (*Short free-running period*; *Sfp-1*, LOD 4.71), Chr 6 (*Sfp-2*, LOD 4.00), Chr8 (*Sfp-3*, LOD 4.95) and Chr 11 (*Sfp-4*, LOD 4.84) (Fig. 3B, Table 2). The one LOD confidence intervals of *Arrh-1* and *Sfp-3* on Chr 8 were observed to overlap (Fig. S3).

**Table 2:** Summary of QTLs affecting the circadian ratio and free-running period.

QTL	Nearest marker (cM)^a^	Peak LOD score^b^	Peak position (cM)	% variance^c^	Phenotype at the nearest locus (%)±S.E.M			*p*-value^d^
Circadian ratio					C/C	B/C	B/B	
-	*D3Mit38* (70.3)	2.63*	70.3	5	59.13±3.62	69.68±2.16	73.47±2.61	<0.01
*Arrh-1*	*D8Mit9* (33.5)	5.26**	33.5	10	54.53±3.70	68.75±2.05	76.09±2.70	<0.0001

QTL	Nearest marker (cM)	Peak LOD score	Peak position (cM)	% variance	Phenotype at the nearest locus (hours)±S.E.M			*p*-value
Free-running period					C/C	B/C	B/B	
*Sfp-1*	*D1Mit71* (19.5)	4.71**	22.3	9	22.53±0.07	22.79±0.06	23.05±0.09	<0.0001
*Sfp-2*	*D6Mit188* (32.5)	4.00**	32.5	8	22.48±0.08	22.80±0.06	22.99±0.09	<0.001
*Sfp-3*	*D8Mit7* (32.0)	4.95**	32.0	10	22.50±0.08	22.72±0.06	23.07±0.08	<0.0001
*Sfp-4*	*D11Mit179* (52.0)	4.84**	52.0	10	22.53±0.08	22.73±0.06	23.09±0.09	<0.0001

a Map position is based on the Mouse Genome Database.

b Genome-wide 5% (**), and 10% (*) level estimated by 1000 runs of a permutation test for each phenotype.

c Percentage of the phenotypic variance accounted for by each QTL.

d Estimated by one-way ANOVA analysis.

**Figure 4:**
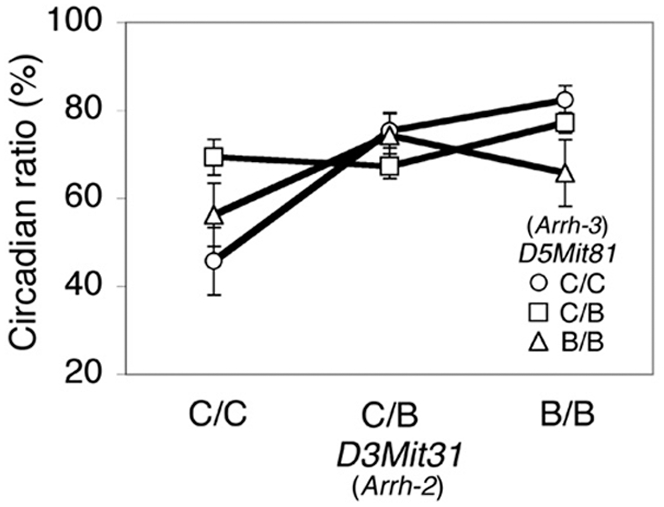
Epistatic interaction between D3Mit31(Arrh-2) and D5Mit81 (Arrh-3) for the circadian ratio. Open circles and open squares represent the homozygous and heterozygous circadian variant (C) alleles for *D5Mit81* (*Arrh-3*), respectively. Open triangles represent the homozygous C57BL/6 (B) allele. Three genotypes for the *D3Mit31* (*Arrh-2*) locus are shown on the x-axis. Each value represents the mean±S.E.M.

**Figure 5:**
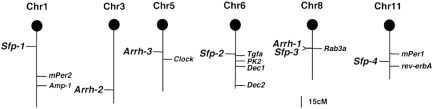
Chromosomal locations of QTLs, and clock and clock-related genes. Each vertical bar represents a mouse chromosome. Black circles represent the centromeres. Symbols on the left side of each chromosome are the QTLs detected in the present study. Symbols on the right side of each chromosome are the known clock and clock-related genes including previously mapped QTLs [5].

To detect epistatic interaction, we performed two-locus interaction analysis for all possible markers using Map Manager QTXb20. If the overall effect exceeded the genome-wide 5% significance level, the interaction effect was tested using approximate genome-wide thresholds. As a result, significant epistatic QTLs (*p* = 0.0011) between *D3Mit31* (*Arrh-2*) and *D5Mit81* (*Arrh-3*) were detected for the circadian ratio (Fig. 4). As shown in Fig. 4, if the genotypes for *D3Mit31* and *D5Mit81* were both C/C homozygous, the mean circadian ratio was 45.74 (±7.65, S.E.M), which was lower than the other combinations of genotype. The QTLs determined in this study, and the clock and clock-related genes are summarized in Fig. 5.

### Sequence of the candidate genes

To search the candidate genes for *Arrh-1* and *Sfp-3* on Chr 8, we sequenced the coding regions of the genes mapped between *D8Mit205* (30.0 cM, 52.3 Mb) and *D8Mit249* (37.0 cM, 83.8 Mb) that covered one LOD support interval of *Arrh-1* and *Sfp-3*. Among the genes located in this region, we selected 69 genes that are highly or moderately expressed in the SCN (H.R. Ueda, unpublished data, Table S2). When compared with C57BL/6J, 7 genes (*Spcs3*, *Wdr17*, *Npy1r*, *Psd3*, *Gdf1*, *Fcho1*, and *Eps15l1*) in the circadian variant were found to have base substitutions causing amino acid changes (Table 3). The sequences of the 7 genes in the CASP mice were the same as those in the C57BL/6J mice.

**Table 3:** Mutations with amino acids substitution in the circadian variant.

Gene name	Position (Mb)	Mutation	Amino acids substitution
*Spcs3*	56.0	495T→A	His165Gln
*Wdr17*	56.1	1732A→T	Thr578Ser
*Npy1r*	69.6	361G→A	Ile121Val
*Psd3*	70.6	80C→T	Ala7Val
*Gdf1*	73.2	332C→T	Ser111Leu
		520T→A	Val177Glu
*Fcho1*	74.6	1520T→C	Leu507Pro
*Eps15l1*	75.3	2456G→A	Ser819Asn

### Clock gene expression in the SCN of the circadian variant under LD conditions

**Figure 6:**
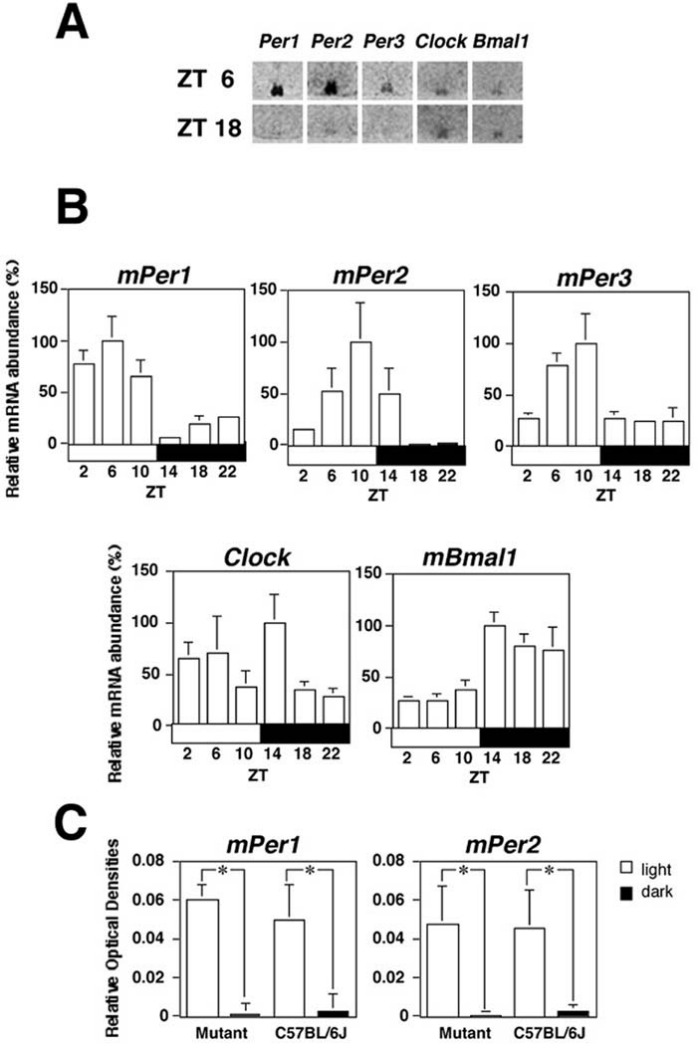
Expression of clock genes in the SCN of the circadian variant mice. (A) Representative autoradiographs of *mPer1*, *mPer2*, *mPer3*, *Clock*, and *Bmal1* signal at ZT 6 and 18 in arrhythmic circadian variant mice. (B) Temporal changes of expression of clock genes in arrhythmic circadian variant mice (mean values±S.E.M., *n* = 4–5). The white and black bars represent the light and dark, respectively. (C) Light-induced expression of *mPer1* and *mPer2* in the circadian variants and C57BL/6J mice (mean values±S.E.M., *n* = 3–4, * *p*<0.05, Mann-Whitney *U* test).

The SCN is known to be a circadian pacemaker controlling behavioral rhythms. Therefore, we studied the expression of known clock genes (*mPer1*, *mPer2*, *mPer3*, *Clock*, and *mBmal1*) and the photic induction of *mPer1* and *mPer2* genes in the SCN of the circadian variant mice (Fig. 6A, B, C). The levels of all the *mPer* genes were high during the light and low during the dark with a peak at zeitgeber time (ZT) 6 for *mPer1* and ZT10 for *mPer2* and *mPer3*. In contrast, the level of *mBmal1* increased during the dark and decreased during the light. *Clock* gene expression did not exhibit clear daily rhythmicity. Under DD conditions when behavioral rhythms persisted, the same patterns as those of LD 12∶12 were observed in all genes (data not shown). A 15-min light pulse induced *mPer1* and *mPer2* expression in these mice in a manner similar to that in the C57BL/6J mice (Fig. 6C).

### Real-time monitoring analysis of mPer1::luc bioluminescence rhythms in the SCN

**Figure 7:**
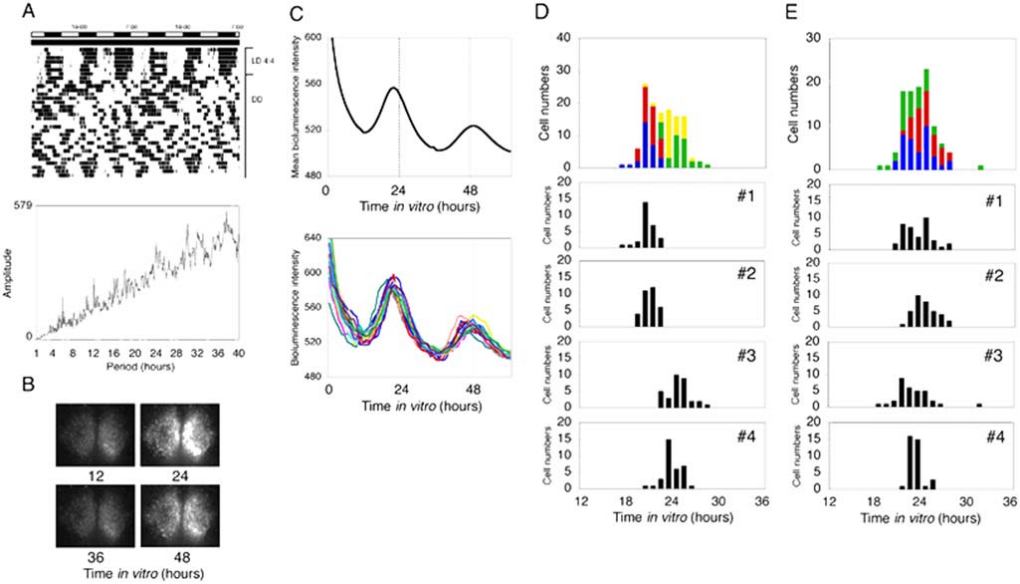
Real-time analysis of mPer1::luc bioluminescence in the SCN of arrhythmic circadian variant mice. (A) Representative activity profile and the result of χ^2^ periodgram analysis for the final 10 days. (B) Images of *mPer1::luc* bioluminescence in the SCN of the arrhythmic mouse shown in (A). The number below each image represents time after SCN slice preparation. (C) Temporal profiles of *mPer1::luc* bioluminescence obtained from the whole SCN (sum of the right and left SCN) (above) and 20 SCN neurons (below) of the arrhythmic mouse shown in (A). The x-axis represents time (hours) after culture initiation and the y-axis is the intensity of *mPer1::luc* bioluminescence. Peak time distributions of individual SCN neurons of arrhythmic mice prepared at 1700–1800 (D) and 0500–0600 (E). The temporal location of the first peak (from 12 h to 36 h *in vitro*) was examined in each SCN neuron. Peak time distributions of each mouse (#1–4) and the piled data (the top graph) are shown.

Since the *in situ* hybridization study described above was unable to reveal the temporal changes of clock gene expression in the arrhythmic mice in which phase references were not determined under DD, we performed real-time monitoring analysis of *mPer1::luc* bioluminescence rhythms of the SCN in which activity of an individual SCN can be traced. Contrary to our expectation, *mPer1::luc* bioluminescence in the SCN, regardless of a single neuron or its population, exhibited clear circadian fluctuation (Fig. 7A, B, C) in all the arrhythmic mice examined. If the SCN maintains the circadian rhythm in spite of the fact that the mice are behaviorally arrhythmic, it might be expected that the phases of the bioluminescence rhythm in the SCN sampled at different times would not coincide. However, the peak time for slices sampled 12 h apart (1700–1800, 0500–0600) appeared approximately 23 h (2300±0055 h for 1700–1800, 2330±0029 h for 0500–0600) after the preparation (Fig. 7D, E).

## Discussion

In the present study, genetic screens were carried out on the progeny of wild-caught *castaneus* mice. One reason for selecting the *castaneus* mice is their high percentage of polymorphic genetic markers, such as microsatellites, which is considerably useful for genetic mapping. A second reason is that the F_1_ progeny (both sexes) of *castaneus* mice and laboratory mice (*Mus musculus domesticus*), such as C57BL, are fertile, which enables us to use an F_2_ intercross for genetic mapping [27]. Finally, we reasoned that natural populations of organisms conserve genetic diversity and many naturally occurring variant genes are maintained within a wild mouse population. In the absence of gene interactions, the variant genes are assumed to have subtle effects on the phenotype. However, these variants are important in considering the function of a highly complex system since many common human diseases, such as diabetes, hypertension and alcoholism, are caused by naturally occurring variant genes and their interactions. Certainly, induced mutations such as *Clock* are important for understanding the mechanisms of the circadian clock [28]; however, our approach to isolate naturally occurring mutations that cannot be achieved by mutagenesis or gene knockout may be useful for understanding the mechanisms underlying circadian and its related disorders in human [29].

In this study, we detected QTLs with main effects affecting the circadian ratio (*Arrh-1*) and τ (*Sfp-1*, *2*, *3*, *4*). Of these QTLs, one LOD confidence interval for *Arrh-1* and that for *Sfp-3* overlapped, suggesting that the same gene might contribute to these phenotypes. With regard to the circadian ratio, the effects of epistatic interactions, which were not detected as main effects, were observed between Chr 3 (*Arrh-2*) and Chr 5 (*Arrh-3*), indicating that circadian rhythms are sustained by the complex interaction among multiple genes.

When we compared *Arrh-1* with the mapped loci of known clock-related genes, we found that *Arrh-1* was closely mapped to the position of the *Rab3a* gene, which has been reported to affect τ; a point mutation in the conserved amino acid (Asp77Gly) within the GTP-binding domain of the Rab3a protein shortened the circadian period of locomotor activity [30]. Although we analyzed the sequence of the open reading frame and the expression level of *Rab3a* mRNA in the SCN, no difference was detected between the circadian variant and the C57BL/6J mice. Instead, we found that 7 genes (*Spcs3*, *Wdr17*, *Npy1r*, *Psd3*, *Gdf1*, *Fcho1*, and *Eps15l1*) had base substitutions causing amino acid changes in the circadian variant. Among these genes, *Npy1r* (neuropeptide Y receptor Y1) might be an interesting candidate, since the mice lacking *Npy1r* have been reported to exhibit reduced locomotor activity [31]. Other reports also indicated that NPY Y1 receptors are involved to some degree in light-induced phase shifts [32], [33]. In addition, the region in which we detected amino acid substitution in the circadian variant is well conserved among mice, rats, dogs, and humans. Another potential candidate is the *Gdf1* gene. *Gdf1* is known to regulate left-right patterning during development in mice. Although the function in the adult animal has not been determined [34], *Gdf1* mRNA is highly expressed in the SCN (online information in the Allen Brain Atlas; www.brain-map.org/welcome.do), raising the possibility that this gene is related to clock functions in the SCN.

Compared with the previous QTL analysis for τ, the mapped positions of *Sfp-1*, *2*, *3* and *4* were completely different [4]. We also found that *Tgfa* (35.8 cM) and *Prok2* (41.0 cM) on Chr 6, and *rev-erbA* (57.0 cM) on Chr 11 were close to the map positions of *Sfp-2* and *Sfp-4*, respectively [10], [35]–[37]. However, there were no differences in the amino acid sequences of these genes between the circadian variant and the C57BL/6J mice. Thus, we now determined the multiple QTLs that were not identified in previous studies using the established laboratory mouse strains.

In many mice exhibiting abnormal circadian behavioral rhythms, the expression of clock genes is severely affected in the SCN [15], [38]–[44]. However, compared with previous studies, *in situ* analysis of our mice revealed no abnormal patterns of clock gene expression in the SCN when behavioral rhythms persisted under LD 12∶12 and DD conditions [39], [40], [45]. In addition, no obvious change in photic induction of *mPer1* and *mPer2*, or in the gross anatomy (Nissl stain) of the SCN was found. To overcome the limitations of *in situ* methods for monitoring circadian rhythms of each individual SCN, we performed real-time monitoring analysis using the *mPer1::luc* reporter system [46], [47]. Unexpectedly, *mPer1::luc* bioluminescence of an individual SCN neuron or its population exhibited circadian fluctuations despite behavioral arrhythmicity. For this result, there are two possible explanations; either the SCN oscillation continued, which was not reflected in behavioral rhythms by impaired output pathway, or desynchronized SCN neurons were reset by the stimulation of the culture preparation (e.g. the stimulation of decapitation, slicing the brain, or placing the slice on culture membranes etc.). To assess these possibilities, we compared the bioluminescence rhythm sampled 12 h out of phase. The results indicated that the peak time of *mPer1::luc* rhythms was dependent on the preparation time and appeared almost 23 h after the preparation in both cases. These results suggested that circadian oscillation of individual SCN neurons might be reset and synchronized by culture stimulation. This is consistent with the behavioral data demonstrating the restoration of circadian rhythms by a light pulse given to arrhythmic mice (Fig. 1I) [48]. Our results appear to be comparable with Yoshikawa et al. [49] who demonstrated that the peak time of SCN bioluminescence rhythms was completely determined by the time of the culture preparation in constant light-induced arrhythmic rats. Although it is possible that behavioral arrhythmicity in our mice results from desynchronization among individual SCN neurons, this does not eliminate another possibility that the preparation stimulation itself regenerates the circadian oscillation of the SCN. In any case, our finding that arrhythmic behavior may not be ascribed to core circadian mechanisms will provide an important insight into the downstream mechanisms that control circadian expression rhythms.

In summary, we isolated a novel variant from a wild mouse population, which abolished behavioral circadian rhythms under DD, and mapped the positions of the multiple QTLs affecting the persistence of circadian rhythmicity and τ. The analysis of clock gene expression in the SCN revealed that the dysfunction in this variant was possibly due to desynchronizing mechanisms among the SCN neurons. Although further studies are required to identify the genes responsible for this dysfunction and define the mechanisms underlying the desynchronization, our variants arising from the interaction between multiple genetic variants may prove useful in understanding the complex circadian system and the mechanisms underlying circadian and its related disorders in human.

## Supporting Information

Table S1 Microsatellite markers genotyped and their map position (cM).
(0.17 MB DOC)

Table S2 The list of genes examined the coding sequence.
(0.06 MB DOC)

Figure S1 The genomic constitution of the parental variant male mouse. Grey arrow heads with numbers show Mit markers used for typing of F2 mice (total 104 markers), and black arrow heads for that of the parental variant male mouse. C/C: homozygous allele for circadian variant, C/B: heterozygous allele, B/B: homozygous allele for C57BL/6J.
(1.01 MB TIF)

Figure S2 Correlation of phenotypic values of the circadian ratio (x-axis) and free-running period (y-axis) in F2 mice. Significant correlation was observed (y = 22.471+0.005×, ñ = 0.198, p = 0.003 by Spearman's test).
(0.57 MB TIF)

Figure S3 Overlapping of Arrh-1 and Sfp-3 on Chr 8. Overlay of LOD score plots for the circadian ratio (black line) and free-running period (gray line). 1-LOD support intervals are shown for each phenotype.
(4.19 MB TIF)
