# Supplementary material for: Genetic and Molecular Analysis of Wild-Derived Arrhythmic Mice
Source: PLoS One. 2009 Jan 28;4(1):e4301. doi: 10.1371/journal.pone.0004301 (PMC2628734; doi:10.1371/journal.pone.0004301)
Supplement: Table S1 — Microsatellite markers genotyped and their map position (cM). (0.17 MB DOC) [file pone.0004301.s001.doc]

Supplemental Table 1. Microsatellite markers genotyped and their map position (cM).

| Marker | Map  Position | Marker | Map  Position | Marker | Map  Position | Marker | Map  Position |
| --- | --- | --- | --- | --- | --- | --- | --- |
| D1Mit294 | 8.5 | D3Mit142 | 50.4 | *D6Mit77* | 15.8 | *D9Mit206* | 20.0 |
| *D1Mit119* | 10.0 | *D3Mit215* | 55.0 | *D6Mit224* | 20.2 | *D9Mit1006* | (51Mbp) |
| *D1Mit432* | 15.0 | *D3Mit42* | 55.8 | *D6Mit185* | 27.0 | *D9Mit191* | 26.0 |
| *D1Mit71* | 19.5 | *D3Mit13* | 61.8 | *D6Mit188* | 32.5 | *D9Mit248* | 32.0 |
| *D1Mit174* | 24.7 | *D3Mit 194* | 67.6 | *D6Mit5* | 34.0 | *D9Mit207* | 33.0 |
| *D1Mit236* | 25.7 | *D3Mit196* | 69.2 | *D6Mit215* | 41.5 | *D9Mit107* | 40.0 |
| *D1Mit1000* | (65Mbp) | *D3Mit38* | 70.3 | *D6Mit63* | 49.7 | *D9Mit11* | 48.0 |
| *D1Mit303* | 34.8 | *D3Mit197* | 71.8 | *D6Mit256* | 60.9 | *D9Mit198* | 49.0 |
| *D1Mit46* | 43.1 | *D3Mit292* | 72.9 | *D6Mit14* | 71.2 | *D9Mit136* | 54.0 |
| *D1Mit49* | 54.4 | *D3Mit31* | 76.2 |  |  | *D9Mit17* | 62.0 |
| *D1Mit10* | 56.6 | *D3Mit147* | 79.4 | *D7Mit74* | 2.0 | *D9Mit215* | 63.0 |
| *D1Mit97* | 65.0 | *D3Mit32* | 80.2 | *D7Mit76* | 3.4 | *D9Mit52* | 72.0 |
| *D1Mit159* | 81.6 | *D3Mit219* | 84.9 | *D7Mit22* | 8.0 | *D9Mit152* | 73.0 |
| *D1Mit314* | 86.6 |  |  | *D7Mit228* | 18.0 |  |  |
| *D1Mit36* | 92.3 | *D4Mit106* | 6.3 | *D7Mit69* | 24.5 | *D10Mit77* | 4.0(9.0Mbp) |
| *D1Mit402* | 92.3 | *D4Mit138* | 17.9 | *D7Mit70* | 27.8 | *D10Mit123* | 4.0(9.9Mbp) |
| *D1Mit540* | 95.8 | *D4Mit111* | 21.9 | *D7Mit346* | 34.0 | *D10Mit169* | 11.0 |
| *D1Mit152* | 101.5 | *D4Mit139* | 28.6 | *D7Mit146* | 37.0 | *D10Mit184* | 18.6 |
| *D1Mit293* | 109.6 | *D4Mit326* | 31.0 | *D7Mit220* | 52.4 | *D10Mit31* | 36.0 |
|  |  | *D4Mit7* | 35.5 | *D7Mit1002* | (118Mbp) | *D10Mit186* | 40.0 |
| *D2Mit76* | 2.0 | *D4Mit176* | 46.5 | *D7Mit259* | 72.0 | *D10Mit42* | 44.0 |
| *D2Mit2* | 4.0 | *D4Mit155* | 49.6 |  |  | *D10Mit117* | 44.8 |
| *D2Mit149* | 7.0 | *D4Mit199* | 53.6 | *D8Mit141* | 6.0 | *D10Mit95* | 50.3 |
| *D2Mit120* | 17.0 | *D4Mit334* | 57.0 | *D8Mit173* | 11.0 | *D10Mit99* | 60.0 |
| *D2Mit322* | 28.4 | *D4Mit283* | 69.0 | *D8Mit4* | 14.0 | *D10Mit14* | 65.0 |
| *D2Mit327* | 40.4 | *D4Mit190* | 79.0 | *D8Mit24* | 18.0 | *D10Mit35* | 69.0 |
| *D2Mit101* | 52.5 |  |  | *D8Mit5* | 25.0 | *D10Mit236* | 70.0 |
| *D2Mit277* | 69.0 | *D5Mit146* | 1.0 | *D8Mit176* | 26.0 |  |  |
| *D2Mit304* | 73.0 | *D5Mit72* | 9.0 | *D8Mit53* | 29.0 | *D11Mit71* | 1.1 |
| *D2Mit194* | 81.4 | *D5Mit66* | 17.0 | *D8Mit205* | 30.0 | *D11Mit227* | 2.0 |
| *D2Mit55* | 91.0 | *D5Mit389* | 18.0 | *D8Mit7* | 32.0 | *D11Mit229* | 14.0 |
| *D2Mit504* | 91.8 | *D5Mit75* | 20.0 | *D8Mit9* | 33.5 | *D11Mit310* | 24.0 |
| *D2Mit51* | 95.5 | *D5Mit128* | 24.0 | *D8Mit249* | 37.0 | *D11Mit260* | 34.3 |
| *D2Mit52* | 99.0 | *D5Mit78* | 26.0 | *D8Mit248* | 43.0 | *D11Mit7* | 44.3 |
| *D2Mit213* | 105.0 | *D5Mit81* | 28.0 | *D8Mit211* | 49.0 | *D11Mit179* | 52.0 |
| *D2Mit457* | 108.0 | *D5Mit257* | 41.0 | *D8Mit12* | 53.3 | *D11Mit67* | 57.0 |
|  |  | *D5Mit155* | 53.0 | *D8Mit154* | 59.0 | *D11Mit99* | 59.5 |
| *D3Mit60* | 0.0 | *D5Mit210* | 64.0 | *D8Mit13* | 67.0 | *D11Mit330* | 61.0 |
| *D3Mit267* | 6.7 | *D5Mit30* | 72.0 | *D8Mit93* | 72.0 | *D11Mit180* | 66.0 |
| *D3Mit46* | 13.8 | *D5Mit169* | 86.0 |  |  | *D11Mit128* | 68.0 |
| *D3Mit306* | 22.0 |  |  | *D9Mit217* | 4.0 | *D11Mit69* | 71.0 |
| *D3Mit241* | 33.0 | *D6Mit138* | 0.7 | *D9Mit1* | 6.0 | *D11Mit104* | 79.0 |
| *D3Mit22* | 33.7 | *D6Mit116* | 5.5 | *D9Mit64* | 7.0 |  |  |
| *D3Mit29* | 45.2 | *D6Mit48* | 7.5 | *D9Mit90* | 9.0 | *D12Mit1009* | (5Mbp) |

| *D12Mit182* | 2.0 | *D14Mit32* | 32.0 | *D17Mit19* | 3.0 | *D19Mit128* | 10.9 |
| --- | --- | --- | --- | --- | --- | --- | --- |
| *D12Mit11* | 6.0 | *D14Mit67* | 38.0 | *D17Mit213* | 9.3 | *D19Mit85* | 16.0 |
| *D12Mit147* | 16.0 | *D14Mit195* | 44.3 | *D17Mit81* | 16.4 | *D19Mit46* | 24.0 |
| *D12Mit172* | 22.0 | *D14Mit194* | 44.4 | *D17Mit16* | 17.4 | *D19Mit88* | 34.0 |
| *D12Mit201* | 29.0 | *D14Mit75* | 54.0 | *D17Mit52* | 22.9 | *D19Mit53* | 43.0 |
| *D12Mit156* | 34.0 | *D14Mit170* | 63.0 | *D17Mit9* | 29.4 | *D19Mit91* | 47.0 |
| *D12Mit158* | 38.0 |  |  | *D17Mit109* | 34.3 | *D19Mit75* | 52.0 |
| *D12Mit204* | 41.0 | *D15Mit174* | 6.7 | *D17Mit152* | 37.7 | *D19Mit71* | 54.0 |
| *D12Mit194* | 45.0 | *D15Mit10* | 9.9 | *D17Mit127* | 45.3 |  |  |
| *D12Mit97* | 47.0 | *D15Mit11* | 10.4 | *D17Mit189* | 53.3 | *DXMit26* | 1.5 |
| *D12Mit141* | 55.0 | *D15Mit138* | 15.4 | *D17Mit123* | 56.7 | *DXMit137* | 5.7 |
| *D12Mit18* | 58.0 | *D15Mit184* | 25.4 |  |  | *DXMit140* | 19.0 |
|  |  | *D15Mit63* | 29.2 | *D18Mit19* | 2.0 | *DXMit119* | 29.5 |
| *D13Mit158* | 5.0 | *D15Mit187* | 43.6 | *D18Mit30* | 4.0 | *DXMit41* | 39.6 |
| *D13Mit162* | 9.0 | *D15Mit105* | 47.9 | *D18Mit21* | 6.0 | *DXMit95* | 43.0 |
| *D13Mit117* | 19.0 | *D15Mit2* | 46.9 | *D18Mit60* | 16.0 | *DXMit117* | 50.8 |
| *D13Mit139* | 32.0 | *D15Mit33* | 48.6 | *D18Mit89* | 20.0 | *DXMit67* | 60.0 |
| *D13Mit21* | 35.0 | *D15Mit72* | 49.0(84Mbp) | *D18Mit202* | 22.0 | *DXMit135* | 69.0 |
| *D13Mit125* | 44.0 | *D15Mit107* | 49.0(84Mbp) | *D18Mit54* | 26.0 | *DXMit157* | 70.0 |
| *D13Mit191* | 45.0 | *D15Mit190* | 52.8 | *D18Mit90* | 28.0 | *DXMit160* | 73.3 |
| *D13Mit202* | 47.0 | *D15Mit171* | 54.5 | *D18Mit91* | 29.0 |  |  |
| *D13Mit287* | 57.0 | *D15Mit193* | 57.9 | *D18Mit107* | 37.0 |  |  |
| *D13Mit196* | 68.0 |  |  | *D18Mit152* | 44.0 |  |  |
| *D13Mit171* | 71.0 | *D16Mit131* | 4.3 | *D18Mit142* | 47.0 |  |  |
| *D13Mit77* | 73.0 | *D16Mit58* | 23.1 | *D18Mit162* | 50.0 |  |  |
|  |  | *D16Mit48* | 43.3 | *D18Mit3* | 54.0 |  |  |
| *D14Mit147* | 2.5 | *D16Mit202* | 47.3 | *D18Mit25* | 57.0 |  |  |
| *D14Mit44* | 10.0 | *D16Mit49* | 53.0 |  |  |  |  |
| *D14Mit120* | 12.5 | *D16Mit19* | 54.0 | *D19Mit78* | 5.0 |  |  |
| *D14Mit64* | 22.0 | *D16Mit52* | 66.8 | *D19Mit79* | 6.0 |  |  |
| *D14Mit203* | 28.3 | *D16Mit20* | 69.5 | *D19Mit31* | 7.0 |  |  |

NOTE: Map position is based on the Mouse Genome Database.

The *Mit* Markers shaded were used for genotype of F2 mice.
