# Supplementary material for: Genetic and Molecular Analysis of Wild-Derived Arrhythmic Mice
Source: PLoS One. 2009 Jan 28;4(1):e4301. doi: 10.1371/journal.pone.0004301 (PMC2628734; doi:10.1371/journal.pone.0004301)
Supplement: Table S2 — The list of genes examined the coding sequence. (0.06 MB DOC) [file pone.0004301.s002.doc]

Supplemental Table 2. The list of genes examined the coding sequence.

| Gene Name | Position (Mb) | Gene Name | Position (Mb) |
| --- | --- | --- | --- |
| *Spcs3* | 56.0 | Ssbp4 | 73.5 |
| *Wdr17* | 56.1 | *Ifi30* | 73.6 |
| *Fbxo8* | 59.4 | *Jund1* | 73.6 |
| *Hmgb2* | 60.3 | *Pde4c* | 73.6 |
| *Galnt7* | 60.4 | *Rab3a* | 73.6 |
| *Mfap31* | 63.5 | *Ccdc124* | 73.7 |
| *Clcn3* | 63.8 | *Mtapls* | 73.8 |
| *Cbr4* | 64.3 | *Rpl18a* | 73.8 |
| *Spock3* | 65.8 | *Adhd8* | 74.3 |
| *Klhl2* | 67.6 | *Mrpl34* | 74.3 |
| *3110005G23* | 67.8 | *Nr2f6* | 74.3 |
| *March1* | 68.5 | *1500034J01rik* | 74.3 |
| *Npy1r* | 69.6 | *5430437P03rik* | 74.3 |
| *Npy5r* | 69.6 | *1110012M11rik* | 74.4 |
| *Psd3* | 70.6 | *Glt25d1* | 74.5 |
| *4732435N03rik* | 71.2 | *Pgls* | 74.5 |
| *Lpl* | 71.8 | *Fcho1* | 74.6 |
| *Atp6v1b2* | 72.0 | *Rab8a* | 75.0 |
| *Atp13a1* | 72.7 | *Tpm4* | 75.0 |
| *Sf4* | 72.9 | *Ap1m1* | 75.1 |
| *9130404D08rik* | 72.9 | *2510049I19rik* | 75.1 |
| *Cspg3* | 73.0 | *Eps15l1* | 75.2 |
| *Rfxank* | 73.0 | *Calr3* | 75.3 |
| *2310045N01rik* | 73.0 | *Cherp* | 75.3 |
| *Cope* | 73.2 | *9130011J15rik* | 75.4 |
| *Gdf1* | 73.2 | *Sin3b* | 75.6 |
| *Homer3* | 73.2 | *Large* | 75.7 |
| *Comp* | 73.3 | *Hmgb2l1* | 77.8 |
| *Ell* | 73.4 | *Tom1* | 77.9 |
| *Fkbp8* | 73.4 | *Tmem34* | 80.4 |
| *Uba52* | 73.4 | *Rbmxrt* | 81.4 |
| *2810422J05rik* | 73.4 | *D630040G17rik* | 81.9 |
| *5330410G16rik* | 73.4 | *Madh1* | 82.2 |
| *Isyna1* | 73.5 | *Gab1* | 83.6 |
| *Pepep1* | 73.5 |  |  |

NOTE: Map position is based on the Mouse Genome Database.
