# Supplementary figures and images for: Genetic and Molecular Analysis of Wild-Derived Arrhythmic Mice
Source: PLoS One. 2009 Jan 28;4(1):e4301. doi: 10.1371/journal.pone.0004301 (PMC2628734; doi:10.1371/journal.pone.0004301)

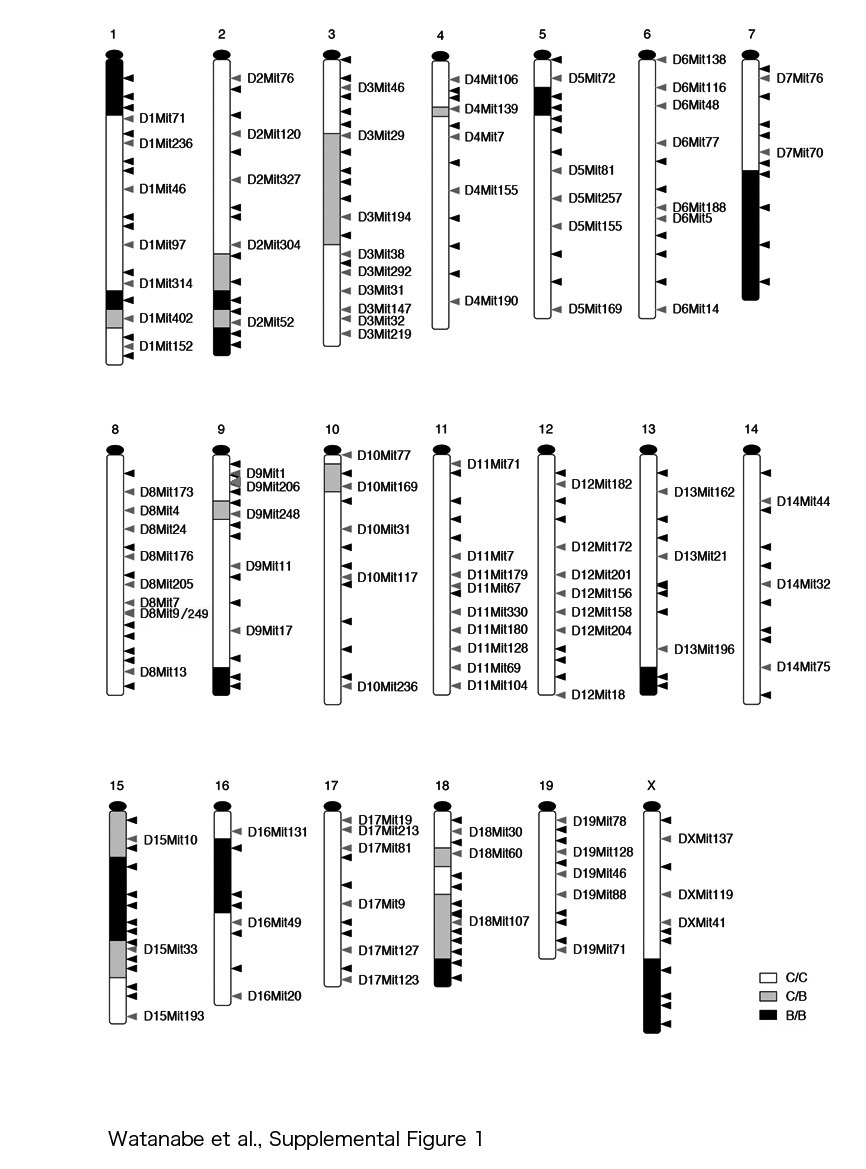

Supplement: Figure S1 — The genomic constitution of the parental variant male mouse. Grey arrow heads with numbers show Mit markers used for typing of F2 mice (total 104 markers), and black arrow heads for that of the parental variant male mouse. C/C: homozygous allele for circadian variant, C/B: heterozygous allele, B/B: homozygous allele for C57BL/6J. (1.01 MB TIF) [file pone.0004301.s003.tif]

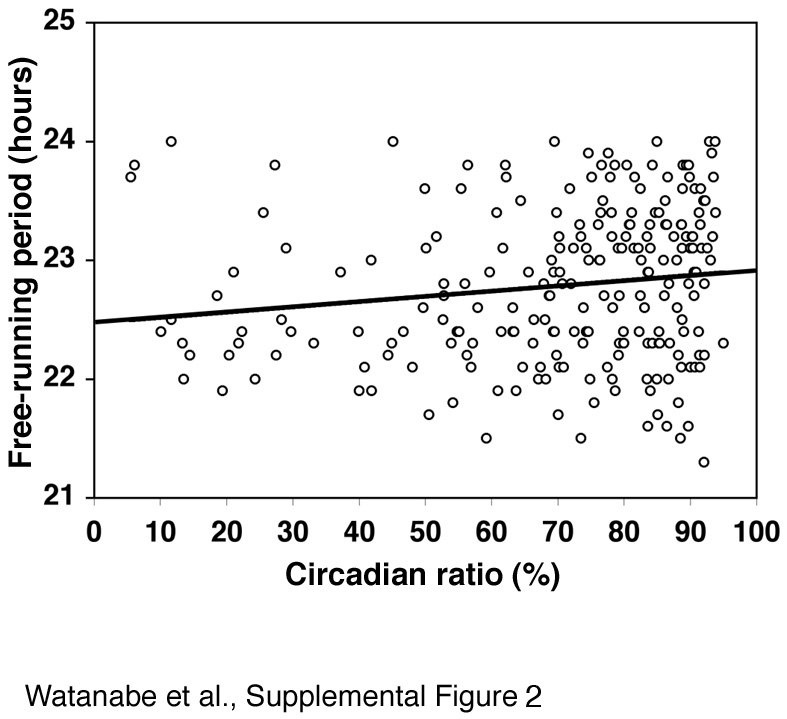

Supplement: Figure S2 — Correlation of phenotypic values of the circadian ratio (x-axis) and free-running period (y-axis) in F2 mice. Significant correlation was observed (y = 22.471+0.005×, ñ = 0.198, p = 0.003 by Spearman's test). (0.57 MB TIF) [file pone.0004301.s004.tif]

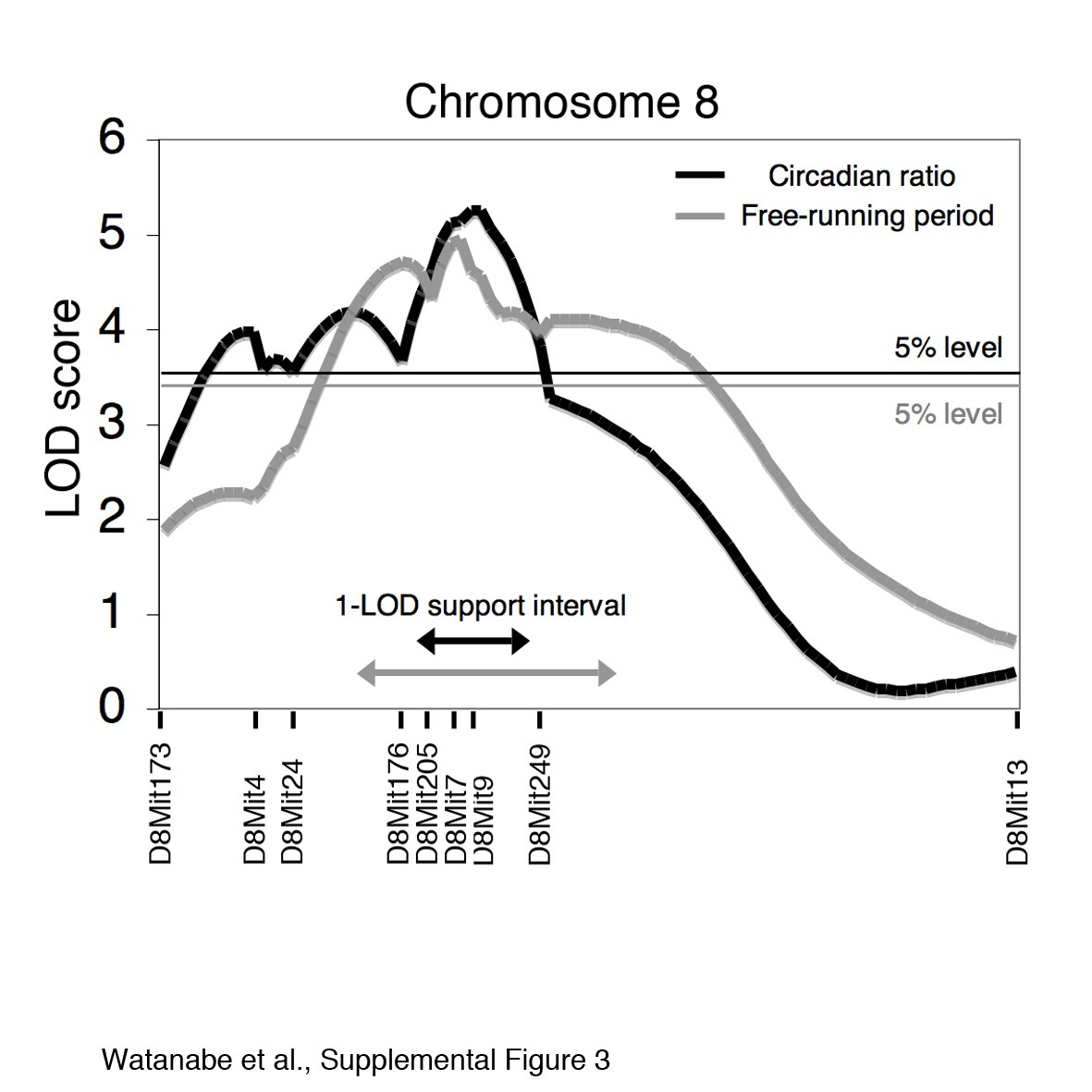

Supplement: Figure S3 — Overlapping of Arrh-1 and Sfp-3 on Chr 8. Overlay of LOD score plots for the circadian ratio (black line) and free-running period (gray line). 1-LOD support intervals are shown for each phenotype. (4.19 MB TIF) [file pone.0004301.s005.tif]
